# Cytokine variations and mood disorders: influence of social stressors and social support

**DOI:** 10.3389/fnins.2014.00416

**Published:** 2014-12-16

**Authors:** Marie-Claude Audet, Robyn J. McQuaid, Zul Merali, Hymie Anisman

**Affiliations:** ^1^Institute of Mental Health ResearchOttawa, ON, Canada; ^2^Department of Neuroscience, Carleton UniversityOttawa, ON, Canada

**Keywords:** aggression, depression, IL - 6, pro-inflammatory cytokines, social status, social stressors, social support, trauma

## Abstract

Stressful events have been implicated in the evolution of mood disorders. In addition to brain neurotransmitters and growth factors, the view has been offered that these disorders might be provoked by the activation of the inflammatory immune system as well as by *de novo* changes of inflammatory cytokines within the brain. The present review describes the impact of social stressors in animals and in humans on behavioral changes reminiscent of depressive states as well as on cytokine functioning. Social stressors increase pro-inflammatory cytokines in circulation as well as in brain regions that have been associated with depression, varying with the animal's social status and/or behavioral methods used to contend with social challenges. Likewise, in humans, social stressors that favor the development of depression are accompanied by elevated circulating cytokine levels and conversely, conditions that limit the cytokine elevations correlated with symptom attenuation or reversal. The implications of these findings are discussed in relation to the potentially powerful effects of social support, social identity, and connectedness in maintaining well-being and in diminishing symptoms of depression.

Without dismissing the importance of other stressful experiences in promoting mood disturbances, particular attention had been devoted to the influence of social stressors in the emergence and continuation of depressive disorders. Indeed, an array of social stressors have been found to act in this capacity, including separation/divorce, unhealthy and unsupportive relationships, loss of a friendship, social rejection, and social alienation (McQuaid et al., 2014a; Slavich and Irwin, 2014). This focus has, in part, stemmed from the frequent findings concerning the importance of social identity and connectedness to psychological well-being (Cruwys et al., 2013, 2014), as well as studies in rodents showing that social challenges have profound and long-lasting effects on neurobiological processes that have been implicated in depressive disorders and adaptation (Chaudhury et al., 2013; Azzinnari et al., 2014; Francis et al., 2014).

In addition to monoamine [e.g., serotonin (5-HT), norepinephine (NE)], neuroendocrine [e.g., corticotropin-releasing hormone (CRH)] and growth factor [e.g., brain-derived neurotrophic factor (BDNF)] variations, it has been suggested that depressive symptoms may stem from activation of the inflammatory immune system, especially that of pro-inflammatory cytokines (e.g., signaling molecules normally released by immune cells in the presence of infectious microorganisms). In this regard, social stressors in rodents, especially those that involve trauma in the form of aggressive social interactions, promoted several neurobiological changes that have been implicated in depression. These have included elevated circulating corticosterone levels (Audet and Anisman, 2010; Audet et al., 2011), increased expression of CRH and its receptors (McQuaid et al., 2013), variations of monoamine turnover and levels (Audet and Anisman, 2010), and changes of neurotrophins such as BDNF (Berton et al., 2006; Fanous et al., 2010) in brain areas that subserve stressor appraisal processes as well as depression [prefrontal cortex (PFC), hippocampus, amygdala, paraventricular nucleus (PVN) of the hypothalamus, and nucleus accumbens]. In addition, social stressors also affected pro-inflammatory cytokines both peripherally and within stress-sensitive brain regions (Bartolomucci et al., 2003; Audet et al., 2010, 2011), supporting the view that activation of these inflammatory markers may contribute to the pathogenesis of stress-related disorders.

The effects elicited by social challenges may depend on the animal's social status or defensive strategies adopted during aggressive interactions. For instance, active coping in the face of aggressive encounters (e.g., active defense, hyperactivity, dominance, aggression) limited or even prevented neuroendocrine and neurochemical effects ordinarily elicited by the social stressor, whereas strategies that involve less resistance (e.g., passive defense, immobility, submissiveness) were related to more profound stressor-induced variations (Bartolomucci, 2007; Audet and Anisman, 2010; Audet et al., 2010; Gómez-Lázaro et al., 2011). These outcomes may have been related to pre-existing neurobiological features associated with genetic dispositions or previous stressor experiences, which might have promoted vulnerability to adverse outcomes in response to social conflicts. The present review delineates the pro-inflammatory impacts of social stressors, with a focus on how these effects in vulnerable individuals may influence the emergence of later mood disturbances and mental health problems, and it is suggested that the positive effects of social support and social connectedness on mood states and well-being may be related to attenuation of pro-inflammatory functioning. Figure 1 describes some of the factors that may moderate the effects of social stressors on cytokine functioning which then promote depressive states.

**Figure 1:**
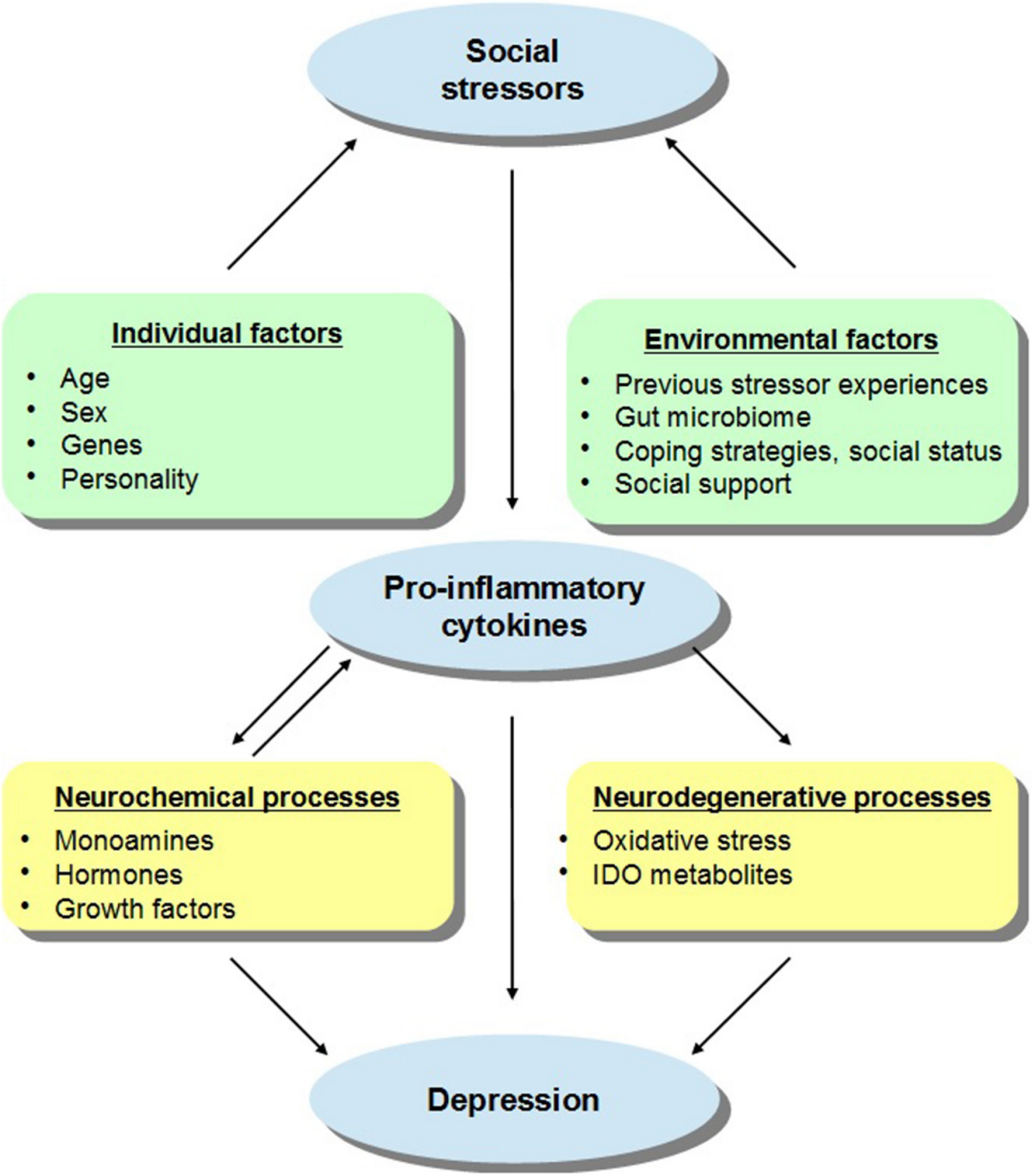
**A schematic representation of the relations between social stressors, pro-inflammatory cytokines, and depressive states, with a particular focus on the individual and environmental factors that may moderate these effects**. It is suggested that in addition to sex and age, the capacity of social stressors to promote inflammatory variations that might lead to depression may be influenced by the presence of genetic and personality factors. For instance, individuals carrying specific gene combinations or polymorphisms (e.g., variants of IL-6, IL-1β, TNF-α) may be more vulnerable to the depressive effects of inflammatory activation provided that they also encounter social stressors. In addition, it is proposed that environmental factors may also impact on stress-related cytokine responses and thus on depressive symptoms. For example, previous stressor experiences in the form of prenatal or early-life adversity or immunological challenges as well as gut bacterial disturbances may influence inflammatory processes and sensitize immune responses to subsequent stressors, thus favoring the emergence of depressive symptoms. However, in the context of adequate coping strategies, higher social status, or in the presence of effective social support, the cytokine effects of stressors may be limited thus buffering against mood disturbances. The activation of pro-inflammatory processes may directly or indirectly influence depressive states. Elevations of cytokines may influence monoamine (e.g., 5-HT, NE), hormone (e.g., CRH), and growth factor (e.g., BDNF) activity which might favor the evolution of depression (and basal hormonal and neurochemical functioning may impact cytokine processes). Alternatively, cytokine variations may stimulate the enzyme indoleamine 2,3-dioxygenase (IDO) and promote the release of neurotoxic metabolites, including kynurenic acid, quinolinic acid, or 3-hydroxykynurenine, and cause oxidative stress, culminating in depression.

## Implications of the inflammatory immune system in depressive illnesses

It has been about two decades since the suggestion was offered that over-activation of the inflammatory immune system might contribute to the pathogenesis of stress-related disorders, especially that of depressive illnesses (Maes, 1995). The inflammatory hypothesis of depression postulated that elevated circulating levels of pro-inflammatory cytokines might promote the evolution and maintenance of depressive symptoms (Maes, 1995, 2008). Meta-analyses have, in fact, indicated that in the absence of infectious pathogens, peripheral concentrations of pro-inflammatory cytokines, especially that of interleukin (IL)-6 and tumor necrosis factor (TNF)-α, were higher in non-medicated individuals with depression than in non-depressed individuals (Dowlati et al., 2010; Liu et al., 2012). Variations of IL-1β in relation to depressive illnesses were less consistently established (Dowlati et al., 2010; Liu et al., 2012), likely because of the difficulty in detecting the very low levels of this cytokine in human circulation. In addition to elevated pro-inflammatory levels, low serum levels of the anti-inflammatory cytokine IL-10 were negatively correlated to depressive symptoms in drug-free depressed individuals (Dhabhar et al., 2009), suggesting that a shift toward a pro-inflammatory state, comprising variations of either or both pro- and anti-inflammatory functioning, may underlie depressive symptoms.

Additional support for the inflammatory hypothesis of depression has come from reports showing that the prevalence of depressive symptoms was relatively elevated in non-medicated patients with chronic inflammatory diseases (e.g., chronic hepatitis C, multiple sclerosis) or acute inflammatory conditions (e.g., surgery, stroke) (Musselman et al., 2001; Cremeans-Smith et al., 2009). As well, immunotherapy with the pro-inflammatory cytokine interferon (IFN)-α for chronic hepatitis C and some types of cancers promoted depressive features (Capuron and Miller, 2004). The latter findings not only support the view that inflammatory factors could play a provocative role in depression, but it appeared that the depressive effects of IFN-α could be attenuated by antidepressant treatments (Raison et al., 2006).

## Social stressors and sterile inflammatory responses

The possible role for pro-inflammatory cytokines in depression has been reinforced by the observation that stressful experiences enhanced inflammatory activity in the absence of infectious pathogens. In this regard, when experienced acutely, social stressor challenges increased circulating concentrations of pro-inflammatory cytokines, especially that of IL-6 and IL-1β (Steptoe et al., 2007). Inflammatory activation has also been reported in individuals chronically exposed to stressful life events, including long-term care of a spouse with dementia (Kiecolt-Glaser et al., 2003), bereavement (Schultze-Florey et al., 2012), prolonged isolation (Yi et al., 2014), effort-reward imbalance at work (Bellingrath et al., 2013), and low socioeconomic status (Gimeno et al., 2007; Loucks et al., 2010). As these stressors may be associated with depression, these data are in keeping with the view that chronic low-grade inflammation resulting from prolonged stressor exposure may be fundamental in the onset of stress-related depressive symptoms.

Among the different stressors that can be experienced, one of the most significant for the emergence of neuropsychiatric symptoms are those involving a violent or traumatic component (e.g., in the form of neglect, maltreatment/abuse, aggression). Based on a meta-analysis, it was concluded that trauma exposure during either childhood or adulthood was positively associated with levels of IL-1β, IL-6, and TNF-α, (but not of IL-2, IL-4, IL-8, or IL-10) and these associations were especially pronounced in individuals who had developed neuropsychiatric symptoms, irrespective of their nature (Tursich et al., 2014). Associations between early-life adversities and higher levels of inflammatory factors have been reported among adults (Danese et al., 2007; Hartwell et al., 2013) but also among children and adolescents (Slopen et al., 2013). Thus, the view was taken that increased inflammatory activity associated with trauma exposure could be initiated shortly after trauma and persist over an extended period of time (or be re-induced, and even exacerbated, upon re-experiencing stressors), possibly progressively fostering sensitivity to later health or mood disturbances. In fact, in addition to having higher baseline cytokine levels, individuals at risk for the development of neuropsychiatric symptoms (owing to previous traumatic experiences) appeared to be more sensitive to the cytokine effects normally induced by a socially stressful experience or to the low-grade inflammation associated with chronic stressors. Higher cytokine levels in individuals with a history of childhood adversities were more pronounced in those currently experiencing high levels of distress, either acutely or on a chronic basis (Carpenter et al., 2010; Kiecolt-Glaser et al., 2011). Thus, the heightened vulnerability to psychiatric conditions in individuals who had been confronted with traumatic events might be related, at least in part, to a fragility or hyper-responsiveness of inflammatory processes resulting from earlier negative experiences (Anisman et al., 2008).

## Peripheral vs. central cytokine processes in depression

The significance of peripheral inflammatory activation in relation to depression is uncertain, especially as mood improvements elicited by medication are not consistently accompanied by a normalization of cytokine levels (Eller et al., 2009; Hannestad et al., 2011). Whereas circulating cytokines may directly or indirectly contribute to the evolution of depressive symptoms, their increased presence in circulation may also be a reflection of the distress experienced by depressed individuals (particularly as depression would itself act as a stressor). Ultimately, however, depression is probably more closely aligned with cytokine variations that occur in the brain or with effects secondary to such changes, including variations of brain neurotransmitters, hormones, or growth factors (see Audet and Anisman, 2013). Several reports also point to the possibility that processes in other organs that share reciprocal connections with the brain, including the microbiota present in the gastrointestinal tract, may also play an important role in this regard (Cryan and Dinan, 2012; Haroon et al., 2012).

A small portion of the pro-inflammatory cytokines released into circulation may access the brain at circumventricular sites (Vitkovic et al., 2000) or through saturable transport systems (Banks, 2006). Thus, cytokine elevations that occur peripherally among stressed and/or depressed individuals might be reflected in the central nervous system, especially as several of the cytokines altered in circulation were also dysregulated in the brain of individuals with depression or with conditions that had been related to and/or are comorbid with the disorder. It is particularly significant, however, that pro-inflammatory cytokines may also be produced endogenously by brain microglia in response to inflammatory or stressor stimuli (Quan et al., 1998; Dantzer et al., 2008; Sukoff Rizzo et al., 2012; Schwartz et al., 2013). The few studies that examined brain cytokine variations in relation to depression revealed that several pro- and anti-inflammatory cytokines (e.g., IL-1α, IL-2, IL-3, IL-5, IL-8, IL-9, IL-10, IL-12A, IL-13, IL-15, IL-18, IFN-γ, and TNF) were up-regulated in post-mortem frontal cortex of depressed patients who had died by natural causes or through suicide (Dean et al., 2010; Shelton et al., 2011). In contrast, in the PVN of the hypothalamus, changes in TNF-α or IL-1β mRNA expression were not observed in depressed patients relative to healthy controls (Wang et al., 2008). A potential role for brain cytokine elevations in depression has also been confirmed in studies using rodent models of the disorder. Social conflicts in male mice increased plasma IL-6 and IL-1β levels and altered mRNA expression of the same cytokines in the PFC and hippocampus (Bartolomucci et al., 2003; Audet et al., 2010, 2011). Moreover, genetic deletion of the IL-1 receptor type 1 on endothelial cells limited brain elevations of IL-1β, TNF-α, and IL-6 mRNA expression elicited by repeated social defeat, indicating that IL-1 signaling likely plays a role in social stress-induced activation of pro-inflammatory cytokines (Wohleb et al., 2014). This is not to say that social stressors are uniquely capable of provoking such changes, as strong physical stressors known to elicit depressive-like behaviors, such as those involving shock and immobilization, increased IL-1β protein and mRNA in serum and in the hypothalamus (O'Connor et al., 2003; Deak et al., 2005) as well as IL-6 in the frontal cortex (Sukoff Rizzo et al., 2012). Likewise, a chronic unpredictable mild stressor regimen that promoted depressive-like features also increased TNF-α levels in the PFC (Liu et al., 2013).

## New players in the cytokine hypothesis of depression

Beyond variations of the traditional pro-inflammatory cytokines IL-6, IL-1β, and TNF-α, depression has been associated with elevations of circulating IL-18, which is involved in cell-mediated immunity (Merendino et al., 2002; Prossin et al., 2011), and macrophage migration inhibitory factor (MIF), which is considered to be a pro-inflammatory cytokine that has neurogenic actions (Musil et al., 2011; Cattaneo et al., 2013). Consistent with these peripheral changes, IL-18 expression in the neocortex increased in subordinate rats after an agonistic encounter (Kroes et al., 2006). As well, genetic deletion of MIF blocked the increased cell proliferation normally elicited by the antidepressant fluoxetine (Conboy et al., 2011), and limited the antidepressant effects and the increased hippocampal BDNF expression ordinarily elicited by exercise (Moon et al., 2012). In addition to pro-inflammatory cytokines, several growth factors including BDNF as well as basic fibroblast growth factor (FGF-2), nerve growth factor (NGF), vascular endothelial growth factor (VEGF), and erythropoietin (EPO) have been implicated in depressive disorders (see Audet and Anisman, 2013 for a detailed description). These growth factors are also influenced by stressors (Cirulli and Alleva, 2009) and as indicated previously (Audet and Anisman, 2013), they may interact with cytokines in affecting behavioral phenotypes.

## Social stressors, cytokines, and depression: does sex matter?

The clinical observation that vulnerability to mood disturbances is more pronounced in women than in men has raised the possibility that males and females might also differ with respect to biological variations that could be linked to depressive symptoms. However, it was reported that females might be more resilient to the cytokine variations associated to depressive states or to stressor exposure. For instance, IL-6 elevations elicited by an acute stressor occurred earlier in men than in women (Edwards et al., 2006). Moreover, elevated levels of IL-6 were apparent in depressed men, but not in depressed women with a late onset of symptoms (Vogelzangs et al., 2012) or suffering from social isolation (Häfner et al., 2011). Animal studies also showed that increased depressive-like behaviors were observed in female but not male IL-10 knockout mice (Mesquita et al., 2008). As well, sensitized IL-1β expression in the PFC and hippocampus apparent after a second stressor exposure in stressed males was not apparent in females (Hudson et al., 2014). Consistent with a role for sex hormones in such outcomes, in females footshocks increased IL-1 expression in the PVN during the diestrus, proestrus, and estrus stages, whereas no elevations were observed during the metestrus stage, suggesting that endogenous progesterone may play a role in the PVN IL-1 response to stressors (Arakawa et al., 2014). This complex picture implies that additional players may contribute to the mood disturbances apparent in females and the question that has to be asked is whether sex hormones (e.g., estrogen, progesterone) play a more provocative role in inflammatory processes than what was initially thought.

## Cytokine variations, aggressiveness, and impulsivity

It is interesting that the inflammatory variations observed among victims of traumatic events are also apparent in individuals perpetrating aggressive or violent acts, and it was suggested that activity of specific cytokines might modulate aggressive and impulsive behaviors. For instance, being physically aggressive during childhood and adolescence predicted reduced baseline levels of pro- (IL-6, IL-1α) and anti-(IL-4, IL-10) inflammatory cytokines in early adulthood (Provençal et al., 2013). Likewise, in mice, peripheral administration of IL-1β suppressed aggressive actions during agonistic encounters (Cirulli et al., 1998). Moreover, among mice that did not express IL-6 the frequency of aggressive postures was elevated and affiliative behaviors were low, whereas overexpression of IL-6 was accompanied by elevated affiliative behaviors (Alleva et al., 1998). Curiously, however, higher plasma IL-6 concentrations in individuals with intermittent explosive disorder were directly correlated with their history of actual aggressive behaviors (Coccaro et al., 2014). Likewise, gene expression of TNF-related cytokines was related to aggression scores in adolescents with bipolar disorder (Barzman et al., 2014). Additional support for a role for inflammatory markers in impulsive or violent behaviors have come from reports showing enhanced levels of pro-inflammatory cytokines among individuals showing deliberate self-harm (Westling et al., 2011), high rage scores (Pesce et al., 2013), or pronounced hostile behaviors (Marsland et al., 2008). Why reduced vs. enhanced cytokine levels were apparent among individuals with aggressive and/or impulsive features in these studies is not entirely clear. Higher levels of childhood maltreatment have been reported in adults with intermittent explosive disorder compared with psychiatric and healthy controls (Fanning et al., 2014). Considering that early-life trauma has been linked to a subsequent increase of inflammatory activity, it might be suggested that the roots and trajectory of aggression (being physically abused vs. being physically aggressive as a child) might have played a role in the different inflammatory patterns observed in these studies.

Consistent with the suggestion that violent and/or impulsive behaviors may be linked to neuropsychiatric conditions, increased IL-1β, IL-6, and TNF-α in post-mortem PFC were observed in teenaged individuals that died by suicide, irrespective of whether they had been diagnosed with major depression (Pandey et al., 2012). Increased protein levels of Toll-like receptors 3 and 4 (associated with increased production of pro-inflammatory cytokines) were also found in post-mortem PFC of depressed adults that had died by suicide compared to that evident in depressed individuals that had died from a cause other than suicide (Pandey et al., 2014). The fact that increased inflammatory activation had been observed in post-mortem brains of suicide individuals is of particular importance, as it raises the possibility that central cytokine variations are linked to suicide rather than depression *per se*. Although suicidal thoughts or ideations may be a symptom of major depression, actual suicide attempts are not necessarily reflective of depressive illnesses, but might be indicative of additional factors, such as impulsivity and extreme or violent behaviors. In this regard, elevated IL-6 levels found in the cerebrospinal fluid of suicidal individuals were more pronounced in those that made efforts of violent suicide (Lindqvist et al., 2009).

## Social trauma: social status and defense strategies matter

It has long been known that social hierarchy, depending on whether the organism is at the top or bottom of the hierarchy, can be particularly stressful and can have marked effects on health and well-being (Sapolsky, 2005). Indeed, the stability of the hierarchy and the availability of resources can have considerable sway on the adaptive neurobiological processes that occur in dominant and submissive/subordinate animals, as can affiliative behaviors of a group, gender, and individual difference factors (personality). Considering that individuals with aggressive profiles may also be vulnerable to neuropsychiatric disorders, the investigation of the potential victim/aggressor dichotomy in relation to inflammatory variations prompted further investigation. Preclinical studies indicated that although aggressive social interactions promoted comparable corticosterone elevations in submissive and dominant mice, some of the cytokine variations elicited were influenced by social status (Audet et al., 2010). Specifically, circulating IL-6 elevations after social conflicts were more pronounced in submissive mice (Audet et al., 2010). As well, higher spleen levels of TNF-α and IL-6 were apparent among mice using passive behaviors (e.g., immobility, decreased reactivity, and low aggression) during aggressive encounters compared to those using active defense strategies (e.g., aggression and territorial control) or to non-stressed controls (Gómez-Lázaro et al., 2011). In contrast, in dominant/winner mice, higher levels of the anti-inflammatory cytokine IL-10 (Stewart et al., 2014) and IL-6 up-regulations were apparent in the PFC (Audet et al., 2010). Together, these findings suggest that active coping strategies during aggressive interactions may be more effective in moderating the cytokine impacts of stressors involving social conflicts than strategies that involve less resistance (Gómez-Lázaro et al., 2011). How these cytokine variations come to be translated into specific behavioral changes is uncertain, nor is it known how chronicity of social stressors will affect cytokine-related disturbances, although it seems that chronic social stressors favor the development of varied pathological conditions (Sapolsky, 2005).

Similar distinctions based on the behavioral strategies used during aggressive encounters in rodents have been reported with respect to other neurochemical markers. In this regard, the increased NE utilization within the PFC and hippocampus observed after social conflicts was more pronounced in submissive than in dominant mice (Audet and Anisman, 2010). As well, latency to escape from an aggressor during a social defeat episode was negatively correlated with BDNF mRNA expression in the hippocampus, suggesting that an active response strategy during stressor exposure might be associated with higher hippocampal BDNF (Arendt et al., 2012). Likewise, depressive-like behaviors and hippocampal BDNF protein reductions were only apparent in those defeated mice that displayed a passive profile during agonistic interactions (Gómez-Lázaro et al., 2011).

## The cytokine hypothesis of depression facing new challenges

There are several unresolved issues pertaining to the relationships between social stressors, pro-inflammatory activation, and depression. Beyond individual differences related to sex, social status, or defense strategies described earlier, whether inflammatory variations and depressive features will emerge as a result of stressor exposure may not always occur and the multiple processes by which this comes about remain to be fully established. For instance, it is uncertain whether a single cytokine is of particular relevance in regard to the provocation of depression, or whether symptoms (or clusters of symptoms) evolve owing to multiple cytokine actions or to interactions between cytokines and other factors. Most studies that examined the relations between circulating cytokines and depression have considered the illness from a broad perspective rather than one that focused on specific symptoms or on depression subtypes. However, it seems that HPA axis and sympathetic nervous system activity (Gold and Chrousos, 2002), as well as that of circulating IL-1β and IL-6 (Anisman et al., 1999; Dunjic-Kostic et al., 2013), may vary greatly in individuals with typical vs. atypical features of major depression. In line with this view, a positive correlation between severity of depressive symptoms and IL-6 concentrations was found in melancholic patients, whereas a negative correlation was apparent in depressed patients with atypical features (Karlović et al., 2012). Whether links exist between other cytokines and specific characteristics of the illness remains to be determined, but specific attention needs to be paid to whether inflammatory activation is linked to specific depressive symptoms and not to others.

The fact that traditional antidepressant medication may limit depressive symptoms without affecting pro-inflammatory cytokine activity (Anisman et al., 1999; Eller et al., 2009; Hannestad et al., 2011) and that anti-inflammatory treatments are not systematically effective in limiting depressive symptoms (Köhler et al., 2014), also begs the question as to what the specific contribution of circulating pro-inflammatory cytokines might be in mediating depressive symptoms. Are these cytokines merely peripheral markers of depression or are they involved in the provocation of illness? Are these cytokines reflective of an ongoing biological dysfunction that would inform treatment resistance or vulnerability to relapse? If depression (or most likely specific depressive subtypes) is driven by inflammation, then traditional antidepressants would be expected to improve mood through cytokine-dependent mechanisms, and anti-inflammatory treatments would be effective in attenuating symptoms. However, this would only occur in those patients in whom depressive symptoms were precipitated by inflammatory factors. In this regard, there has been growing acknowledgment of the limited success in defining the genetic and biological complements that are associated with depression, and the suggestion was made that individualized treatment for the illness, based on biologically relevant markers, may be instrumental in guiding treatment selection and enhancing outcomes. Indeed, the identification of specific cytokine markers that might be aligned with particular symptoms or subtypes of depression could be especially useful in the prediction of treatment efficacy.

## New avenues for the inflammatory hypothesis of depression

As already intimated, the processes by which cytokine elevations are elicited by social stressors, and how these come to affect brain functioning, are still not known. In addition to cytokine variations that occur in the brain, neurobiological alterations elicited by stressors at various peripheral sites that interact with brain processes may be relevant to stressor-related disorders, including depression. In this regard, increasing interest has focused on the inflammatory variations that might occur along the gut-brain axis (Cryan and Dinan, 2012; Haroon et al., 2012). It has been suggested that microorganisms that inhabit the gastrointestinal tract, referred to as the gut microbiota, might play an important role in inflammatory responses elicited by stressors. The gut is inhabited by 10^13^–10^14^ commensal bacteria that interact with each other and modulate other systems, including the immune functioning. Alterations in the composition and diversity of the gut microbiota by environmental insults, including stressors, have been correlated with plasma cytokine elevations (Bailey et al., 2011). Moreover, reduction of the gut microbiota by antibiotics prevented the stressor-induced cytokine elevations, indicating that bacteria that live in the gut were necessary for an inflammatory stress response to develop (Bailey et al., 2011). Given that microbiota may have immunoregulatory effects through actions on immune cells, it was suggested that probiotic bacteria (i.e., “good” bacteria that when ingested confer a benefit for the host), by their interactions with commensal gut bacteria, may also influence inflammatory responses elicited by stressors. In support of this view, plasma elevations of pro-inflammatory cytokines provoked by a psychological stressor were prevented in rats treated with the probiotic *Bifidobacterium infantis* (Desbonnet et al., 2010).

Accumulating data have also indicated that the gut microbiota may interact with the central nervous system and that alterations of microbiota composition could modulate brain functions and behaviors (Cryan and Dinan, 2012; Dinan and Cryan, 2012). For example, reduced anxiety-like behaviors as well as altered expression of BDNF and of a variety of NMDA and 5-HT receptors were reported in germ-free mice (i.e., mice that had been maintained in a sterile environment and never been exposed to bacterial microbe) and in mice treated with antibiotics that disrupted gut bacteria (Sudo et al., 2004; Bercik et al., 2011a; Neufeld et al., 2011). Moreover, administration of particular probiotic strains attenuated anxiety and depressive behaviors in rodents (Bravo et al., 2011) and in healthy humans (Messaoudi et al., 2011). As well, ingestion of probiotics in rodents affected central expression of GABA receptor subunits (Bravo et al., 2011), BDNF (Bercik et al., 2011b), and two markers of microglial activation, CD68 and CD11b (Distrutti et al., 2014), although the mechanisms by which this occurred have not been identified.

## Inflammatory gene variants as risk factors for depression and as predictors of treatment response

It has been suggested that inflammatory cytokine activity might serve as a biomarker to predict how individuals cope with stressors, whether they develop depression, and/or whether they respond to different treatment strategies (Anisman et al., 2008; Yoshimura et al., 2009; Cattaneo et al., 2013). Beyond protein or gene expression variations, allelic variants of genes [e.g., in the form of gene polymorphisms, including single-nucleotide polymorphisms (SNPs)] that promote higher transcription of pro-inflammatory cytokines appear to be related to inflammatory variations normally elicited by stressors and in some cases to predict the emergence of depressive symptoms (Bufalino et al., 2013). For instance, cytokine elevations in bereaved individuals were apparent in homozygous carriers of the mutant G allele of the IL-6-174C polymorphism (associated with high IL-6 transcription), but not in those carrying the low transcription C allele (Schultze-Florey et al., 2012). Considering that a positive association between depression and mortality risk has been found in homozygous carriers of the high transcription G allele of the IL-6-174C polymorphism (Cole et al., 2010), it was suggested that the low transcription C allele may protect against physical and mental health problems associated with psychological distress (Schultze-Florey et al., 2012). In support of these findings, depressive symptoms elicited by IFN-α immunotherapy in patients with chronic hepatitis C were reduced in those carrying the C allele of the IL-6-174C polymorphism (Bull et al., 2009). In addition to IL-6, variants of the genes encoding for IL-1β and TNF-α have also been associated with elevated risk for depression (Bufalino et al., 2013). Parenthetically, paralleling the effects of stressors, the depressive effects of immunotherapy were less pronounced among individuals carrying the two long (L/L) alleles of the serotonin transporter gene-linked polymorphic region (5-HTTLPR) relative to individuals carrying the short (S) allele, pointing to the possibility that IFN-α immunotherapy operates like stressors in predicting the development of depression (Bull et al., 2009). Thus, in addition to cytokine sensitivity elicited by external factors (e.g., traumatic events), polymorphic variations on inflammatory genes might also contribute to the vulnerability to social stressors and to the development of depressive illnesses. Whether environmental and genetic influences interact in this regard remains to be investigated, but given the influence of stressors on cytokine functioning, such interactions would be expected.

Just as increased levels of IL-6 (Lanquillon et al., 2000; Yoshimura et al., 2009) as well as up-regulated expression of IL-1β and TNF-α (Cattaneo et al., 2013) predicted a lack of response to different classes of antidepressants, polymorphisms of the IL-1β, IL-6, and IL-11 genes, which were associated with increased cytokine production, were accompanied by a diminished response to antidepressant medication (Uher et al., 2010; Bufalino et al., 2013). Based on these findings, it had been suggested that depressed individuals who repeatedly fail to respond to traditional antidepressants may exhibit a distinct pro-inflammatory profile. This possibility was confirmed in animal studies showing that mice engineered to overexpress IL-6 in the frontal cortex and hippocampus as well as mice that had received intracerebroventricular injections of IL-6 showed a blunted response to the antidepressant effects of fluoxetine (Sukoff Rizzo et al., 2012). In essence, elevated levels of particular cytokines may predict the development of depression, and although IL-6 and TNF-α levels may decline with appropriate treatment (Lanquillon et al., 2000; Yoshimura et al., 2009), if the cytokine levels are too high prior to treatment commencing, possibly reflecting a disturbance of regulatory/inflammatory processes, the effects of antidepressant treatments will be muted. It is equally possible that the elevated cytokine levels that sometimes persist despite positive mood changes elicited by antidepressant treatment may reflect a harbinger for illness recurrence (see Anisman et al., 2008).

## Cytokine variations and positive environments—the good and the bad

If negative events promote cytokine disturbances that undermine well-being, is it the case that positively interpreted events and experiences act against cytokine disturbances and the emergence of depression? Studies in animals have indicated that positive interventions in animal models of depression are associated with reductions of stress-induced cytokine elevations. For instance, increased TNF-α and IL-6 mitogen-stimulated splenocyte production induced by separation from a mom or littermates was attenuated in young piglets that were paired with an aged-matched conspecific (Tuchscherer et al., 2014). As well, in mice, long-term exposure to an enriched environment reduced IL-1β and TNF-α elevations in the hippocampus induced by influenza infection (Jurgens and Johnson, 2012). That said, it has also been shown that environmental enrichment in male mice enhanced the cytokine effects normally elicited by social defeat, probably owing to the aggressive behaviors promoted by enrichment in male mice (McQuaid et al., 2013).

The data derived from human studies have similarly revealed that positive experiences may act against cytokine variations, which could influence mood states. For instance, psychosocial measures of coping and self-esteem were inversely correlated to IL-6 levels (Sjögren et al., 2006) and improvements in coping strategies and resilience after self-administered hypnosis were associated with IL-6 reductions (Schoen and Nowack, 2013). A meta-analysis also demonstrated that mind-body therapies (e.g., Tai Chi, meditation, yoga) tended to reduce IL-6 levels in both healthy and clinical populations (Morgan et al., 2014). Importantly, reductions of depressive symptoms after daily yogic meditation intervention (Black et al., 2013), a mindfulness treatment program (Carlson et al., 2007; Rosenkranz et al., 2013), and cognitive-behavioral therapy (Gazal et al., 2013) were all related to pro-inflammatory cytokine reductions.

Social factors, and particularly having social support, have long been known to diminish some of the adverse effects of stressors. By example, the availability of social support was accompanied by reduced levels of cortisol both in a natural setting and within a laboratory context (Heinrichs et al., 2003). Further, in a stress test the right PFC activation and diminished amygdala activity that were ordinarily elicited could be attenuated by having social support available (Taylor et al., 2008), and having received social support over several days blunted the cortisol response ordinarily elicited by a social stressor and enhanced neuronal activity within the anterior cingulate cortex (Eisenberger et al., 2007). There have similarly been reports showing that social support could influence the plasma cytokine response otherwise elicited by stressors, and could thus influence mood state (Slavich and Irwin, 2014). In fact, it has broadly been reported that among cancer survivors who had social support prior to treatment, later well-being was enhanced. In fact, in the individuals with lower pretreatment social support, the levels of IL-6 increased with illness progression, which predicted the elevation of depressive symptoms (Hughes et al., 2014).

A similar link between social support, cytokines, and general well-being has also been observed among medically healthy individuals. Specifically, low social status was accompanied by elevated IL-6 in response to a stressor (Derry et al., 2013), and social strain emanating from family and friends increased circulating IL-6, whereas having social support modestly protected against this outcome (Yang et al., 2014). Moreover, the rise of IL-6 associated with anger was not evident among individuals who perceived themselves as having high social support (Puterman et al., 2013).

The data available concerning the influence of social stressors and social support on cytokine levels is still limited. Nonetheless, it is certain that social stressors, especially those of an interpersonal nature, such as social rejection and social adversity, can be particularly aversive and it has been suggested that the depressive actions of such experiences may involve activation of pro-inflammatory processes (Slavich and Irwin, 2014). However, it might not always be the case that social support will be prophylactic or remedial in attenuating the effects of stressful experiences, especially if efforts of support are interpreted as being insufficient or actually a reflection of an unsupportive interaction (McQuaid et al., 2014b).

## Concluding comments regarding cytokine involvement in depression and its comorbidities

The specificity of cytokine disturbances to depression has frequently been questioned. In this regard, it was demonstrated that despite the overlap that exists with respect to the symptoms of IFN-α-induced and idiopathic depression, there are differences between the two, leading to the suggestion that cytokines preferentially affect neural circuits associated with psychomotor activity, but have less of an effect on the processes that govern cognitive distortions concerning self-appraisal (Capuron et al., 2009). Furthermore, increased inflammatory activity has been reported in a variety of stress-related disorders, including bipolar disorder (Modabbernia et al., 2013), schizophrenia (Altamura et al., 1999), and post-traumatic stress disorder (Lindqvist et al., 2014), and has been associated with a number of chronic diseases including cancers, heart diseases, metabolic syndrome, diabetes and obesity, auto-immune illnesses, disorders of the digestive system (i.e., inflammatory bowel disease), and neurodegenerative disorders (Anisman et al., 2008). In essence, it is possible that altered cytokine functioning might create a general milieu that favors the development of pathology, but the specific disturbance that is expressed depends on the presence of still other factors being affected. This said, as most of these conditions are often comorbid with depression, it has been suggested that increased inflammation might be a common denominator underlying depressive symptoms across many neuropsychiatric and physical/medical conditions. Indeed, the link between depression and the development of illnesses, such as heart disease, is sufficiently strong to have prompted the suggestion that the presence of depression ought to be viewed as a marker for later physical illnesses (Hayley and Anisman, 2013). Just as stressful events, particularly those that involve social challenges, promote cytokine variations and several pathological conditions, social support has been effective in attenuating these outcomes. Considerable evidence indicates that the effectiveness of support depends on whom the support comes from, and the individual's social identity and social connectedness may be involved in the resolution of depression (Cruwys et al., 2013, 2014). It remains to be determined whether the positive effects of social identity and connectedness in attenuating depression operate through inflammatory processes.

### Conflict of interest statement

The authors declare that the research was conducted in the absence of any commercial or financial relationships that could be construed as a potential conflict of interest.
